# How long can Chinese women work after retirement based on health level: Evidence from the CHARLS

**DOI:** 10.3389/fpubh.2023.987362

**Published:** 2023-02-27

**Authors:** Xiya Cheng, Ya Fang, Yanbing Zeng

**Affiliations:** ^1^Key Laboratory of Health Technology Assessment of Fujian Province, School of Public Health, Xiamen University, Xiamen, China; ^2^School of Public Health, Capital Medical University, Beijing, China

**Keywords:** delayed retirement, women's health, labor participation, Chinese, work capacity

## Abstract

**Objective:**

To further enhance the understanding of factors impacting female participation in the workforce based on health levels and to measure the excess work capacity of middle-aged and older female groups by residence and educational level.

**Methods:**

Data of women aged 45–74 were accessed from the China Health and Retirement Longitudinal Study (CHARLS) from 2011, 2013, 2015, to 2018. The health status of women was comprehensively evaluated by single health variables and frailty index. A Probit model was used to measure the excess working capacity of women by region (rural/urban) and educational level, taking all women aged 45–49, rural women aged 45–49, and rural (illiterate) women in all age groups as the benchmark, respectively.

**Results:**

The excess capacity of all Chinese women aged 50–64 is 1.9 years, and that of women aged 50–74 is 5.1 years. The excess work capacity of women in urban and rural areas and with different educational levels is heterogeneous. The excess working capacity of urban women aged 50–64 is 6.1–7.8 years, and that of urban women aged 50–74 is 9.8–14.9 years. The excess working capacity of urban women aged 50–64 is about 6 times that of rural women. The excess work capacity of highly educated women was 3 times higher than that of illiterate women.

**Conclusion:**

The potential work capacity of Chinese women remains to be exploited, especially for urban and highly educated middle-aged and older women with better conditions of health, whose potential is more significant. A rational retirement policy for women and the progressive implementation of an equal retirement age for men and women will contribute to further advancement of gender equality and healthy aging in the workplace in China.

## 1. Introduction

With decreasing birth rate and increasing life expectancy, numerous countries have initiated reforms attempting to raise the normal retirement age (NRA) in response to tremendous pressure on pension funds (1). China is facing a more severe situation due to early sex ratio imbalance and population expansion. China began to implement the one-child policy in the 1980s, which was continued for nearly 30 years. The long-term one-child policy has led to a persistent low fertility rate, resulting in the formation of hundreds of millions of inverted pyramidal one-child families, which will accelerate China's labor shortage (2). Additionally, according to the World Health Organization, when the aging rate exceeds 7, 14, and 21% it is called “an aging society”, “an aged society,” and “a super-aged society,” respectively. The “aging rate” refers to the proportion of people over 65 years old in a society (3). As China's aging population continues to grow, it is expected that China will become a “super-aged society” by 2035 (4). This labor shortage and aging increase will directly aggravate the pension payment crisis (5). According to statistics, the accumulated amount of China's existing pension fund will be in deficit by the middle of the 21st century without any reform program (6). This suggests that the current pension fund cannot protect the future older population completely. The future sustainability of pension funds will still require government financial support, which will strongly impact China's fiscal sustainability (7). Consequently, in the face of rapid aging and pressure on pension fund payments, raising the normal retirement age is urgent for China.

The retirement age in China is stipulated as 60 for men and 50 for blue-collar women (8). In 1955, the State Council of China promulgated a document raising the retirement age for white-collar women to 55. This gender-specific age policy has been in place ever since (9). The average normal retirement age in Organization of Economic Cooperation and Development (OECD) countries in 2018 was 64.2 years for men and 63.5 years for women (10). Compared with the retirement age regulations of OECD countries, China's retirement age is significantly lower, especially for women. At the same time, more than half of OECD countries have the same retirement age for men and women, and even if the remaining countries adopt the policy of different retirement age for men and women, the retirement age gap between men and women is relatively low. For example, the retirement age gap between men and women in Slovenia is only 0.3 years (11). In contrast, the gap between men and women for retirement age in China is 5 years (for men and white-collar women) or 10 years (for men and blue-collar women)—significantly higher than in OECD countries. Contradictions exist between the relatively elevated life expectancy of Chinese women and the relatively early retirement age. The average life expectancy of Chinese women in 2020 was 79.43 years, while that of men was 73.64 years, according to the *National Bureau of Statistics of China*. Although life expectancy cannot be regarded as an accurate indicator forecasting women's health, it still remains of some indicative significance (12). Although the Chinese government's policies regarding retirement at different ages for men and women were originally intended to protect women's rights, it is undeniable that with China's rapid development and women's increased health and education, these policies have shortened women's careers and hindered their professional development (13).

Under these circumstances, there is still no definite proposal for delaying retirement, even though China initiated research on the formulation and promotion of a delayed retirement policy as early as 2013, but these results are still limited to the academic community (14). Prolonging the retirement age of women may not yield the expected results, not all the older women are capable of working, especially for those burdened by physical or psychosocial health limitations. Simply prolonging the retirement age while disregarding heterogeneity among older women may lead to increased inequality between healthy and unhealthy individuals, further impairing the lives of older women in general (15). Consequently, one of the criteria for determining the delay in retirement age should be whether the older women is able to cope, and for how long, with the excess work (16).

It is well-known that retirement desire increases with age (17). Physical condition of middle-aged and older women tends to decline with age, thus poor physical condition is often cited to explain early retirement (18). As a consequence of the phenomenon, scholars have subsequently introduced other ideas such as economic factors and social security (19, 20). Some scholars believe the impact of health on retirement cannot be ignored even though it is more economical to keep working (21, 22). While numbers of scholars concur with the importance of health as a factor influencing retirement in middle and old age, there are still different arguments on the metrics of health. In studies related to health and retirement, self-rated health was first used as a proxy variable for health (23, 24). However, the endogenous side effects of self-assessed health cannot be avoided, such as the tendency of the unhealthy population to exaggerate their health conditions and retire early on the grounds of illness (25). Thus, objective health indicators have gradually been adopted by scholars to avoid these biases. Mortality, for example, is more serious and has a greater impact on labor force participation behavior (26). Several articles investigate the impacts of specific illnesses on retirement decisions, like chronic disease (27) and disability (28). However, fully objective health measures, although avoiding endogeneity problems, have three drawbacks. First, the dimensionality is single and answers are usually dichotomous variables that do not provide a comprehensive description of the condition (29). Second, the association between objective health status and labor supply is dependent on the individual's occupation, e.g., arthritis affects painters more than white-collar workers (30). Finally, geographical or cultural differences, e.g., the United States and the Netherlands have different criteria for disability (31). Thus, objective ratings of health are prone to measurement bias. To avoid endogeneity problems and measurement bias, Stern combined the two using objective health indicators as instrumental variables for self-rated health (32). Poterba, Venti, and Wise (PVW) combined self-rated health with objective health indicators using principal component analysis to construct a composite health index to comprehensively assess the health of older adults. This index has several important attributes, it has a strong stability over time. It strongly correlates with mortality and is a good predictor of future health events, such as the onset of cancer or diabetes (33, 34). This index has been used in excess work capacity studies in several countries (35–38).

There are two principal approaches to health-level flexible retirement age measurements. First, based on mortality, Wise estimates excess work capacity by comparing decline in mortality in the long-term group (as a proxy for health improvement) with variations in labor participation among the older population, but this does not provide a reasonable explanation for the rising trend in female employment rates over time (39). The second approach by Cutler, Meara, and Richards-Shubik (CMR) is based on comprehensive health status, which associates employment rate with health status by using the regression coefficient of the middle-aged group to calculate the expected labor participation rate of the older group. The difference between the expected rate and the actual rate referred to the excess working capacity (40), the approach has been widely adopted in countries such as Spain (38), Japan (41), and Denmark (36).

From a health perspective, to what age can Chinese women delay retirement? Since China has not formally implemented a delayed retirement policy, answering this question can help promote the delayed retirement policy (13). Therefore, this paper uses the CMR health measurement model, combined with the PVW comprehensive health index, to measure women's excess work capacity based on their health level, and to a certain extent, to simulate and evaluate whether Chinese women have the health capacity to support their delayed retirement. This paper further enriches the measurement of retirement age, and provides theoretical support for the delayed retirement policy.

## 2. Materials and methods

### 2.1. Data

Data for this study were collected from four periods of the China Health and Retirement Longitudinal Study (CHARLS) in 2011, 2013, 2015, and 2018. CHARLS is a nationwide questionnaire survey conducted in 150 counties among 28 provinces (autonomous regions and municipalities) in China (42), created by the National Development Research Institute of Peking University. The CHARLS baseline survey was chosen because it contains a large number of health status measures, and it focuses on the Chinese population over the age of 45, which matches the required sample age for this study. Basic information such as health status and function, health care and insurance, and work retirement pension from the CHARLS questionnaire were involved in this study. Women aged 45–74 were selected as subjects, those missing critical variables were excluded. Eventually, a valid sample of 31 937 cases was obtained, including 19 524 cases in the labor participation group and 12,413 cases in the non-labor participation group. As one person may appear in multiple waves, standard errors were clustered at the individual level.

### 2.2. Variables

#### 2.2.1. Labor participation

In this study, labor participation is defined as the dependent variable and is a binary dummy variable. If labor participation behaviors exist, it was recorded as 1, and if not, as 0. Labor participation in CHARLS consists of being engaged in agricultural production activities for more than 10 days during the past year, being engaged in paid work for at least 1-h last week, and being currently on leave or training status. Those who had never worked in their lifetime were excluded from this study (*n* = 1996).

#### 2.2.2. Women's health

In this paper, two main forms of health expressions are adopted, the first one is the inclusion of single health variables, including subjective ratings and objective measures. The subjective assessment is the self-assessment of health status, which is divided into five levels: very good, good, fair, poor, and very poor. Objective measures include the depression (CESD-10) score, limitations on activities of daily living (ADLs), instrumental activities of daily living (IADLs), disabilities, psychiatric conditions, eyesight, hearing, chronic falls, fractures, smoking, and drinking. In CHARLS, depression was assessed using the Center for Epidemiologic Studies Depression Scale (CESD-10), ADLs are measured using a 6-item summary assessed with an ADL scale that includes eating, dressing, transferring, bathing, using the toilet, and continence, IADLs cover telephone, housework, cooking, medication, shopping, and financial management, with four different options for each measure: “no difficulty, difficulty but can still do it, difficulty needing help, and unable to do it” (42). In this study, having any ADL limitation was identified as an ADL disability, and IADL was classified in the same way. The second is the use of a composite health index instead of a series of health indicators, drawing primarily from Poterba, Venti, and Wise's composite health index (hereafter referred to as the PVW index) (34). This health index was constructed based on 19 questions, including self-rated health, functional limitations, hospital admissions, and other health indicators. The first principal component of the indicator set is used first and is ranked in percentile order based on principal component scores, so that the index is a percentile scale from 1 to 100, with higher scores associated with better health. The weights of the components in constructing the PVW index are shown in Appendix Table A1.

#### 2.2.3. Control variables

Sociodemographic characteristics constituted the control variables for this study. The main ones included age, place of residence, marital status, educational attainment, and health insurance. Age was transformed into a categorical variable according to the needs of the study, and age 45–74 years was assigned as a group every 5 years, for a total of 6 groups. Marital status was a dummy variable, coded as 1 and 0, representing in marriage and not in marriage, respectively. Considering the differences in educational background over time, an education of junior high school and above was considered as higher education (43). Therefore, education level was divided into illiterate, elementary school, middle school, and above. Health insurance coverage was a dichotomous indicator of whether the participant reported having any type of health insurance. See Appendix Table A2 for variable definitions and codes.

### 2.3. Statistical analysis

#### 2.3.1. Benchmark group setting

The key to the CMR estimation method is to assume that different age groups in the same health state have the same working capacity. Therefore, a younger group was selected in the study as reference for calculating the excess work capacity of the older group in the same health state. Women in this age group are farther away from retirement and retirement decisions tend to be independent of the pension system during this time, thus the effect of health characteristics on labor participation could be accurately assessed. However, there is a natural benchmark group in China, unlike in developed countries, namely, Chinese rural women. More than 90% of the older people in rural China are primarily engaged in agricultural activities and do not require a clear retirement age as in urban employment (44). They usually do not choose to retire as long as they are physically able to continue working (13). There is a natural advantage in using this benchmark group as comparison to the younger group. Consequently, three benchmark groups are established in this paper: first, all women aged 45–49 years to predict the excess work capacity of all women aged 50–74 with rural and urban subgroups; second, rural women aged 45–49 to predict the capacity of urban women aged over 50 years; and third, rural women aged 50–54, 55–59, 60–64, 65–69, and 70–74 to predict the capacity of urban women with the same age.

This paper estimates the excess working capacity of older women through two steps. In the first step, a Probit model was used to estimate the relationship between health and labor participation in the benchmark group to obtain coefficients. In the second step, the coefficients obtained in the first step were combined with actual health characteristics of the post-retirement age group to predict the proportion of older workers (women aged 50–64 and women aged 50–74) working, and then to calculate their excess working capacity against the actual working proportion. In this sense, the excess work capacity defined in this paper refers to the ability to work that is determined by physical health.

Benchmark regression models of this paper were set as follows.


(1) $$\begin{array}{l} {Work}_{i}{=\beta }_{0}{+\beta }_{1}{Health}_{ij}{+\beta }_{2}X_{ik}+\varepsilon_{i} \end{array}$$

In model 1, *Work*_*i*_ represents individual labor participation status. *Health*_*ij*_(j = 1, 2…8) represents individual health characteristics, including a series of health indicators like self-assessment of health, chronic diseases, and disability; X_*k*_ (*k* = 1, 2, 3, 4) represents control variables; β_0_ is a constant term, β_1_ is a health coefficient, and ε_i_ is a random disturbance term.


(2) $$\begin{array}{l} {Work}_{i}{=\beta }_{0}{+\beta }_{1}{PVW}_{i}{+\beta }_{2}X_{ik}+\varepsilon_{i} \end{array}$$

In model 2, the health index was not regressed on multiple sets of health indicators but rather a percentile ranking after converting all health indicators of an individual into a PVW index, which is done to corroborate the validity of model 1. Higher index is associated with better health, which means that the health coefficient can be interpreted as the effect of a one percentage point change in the health distribution on the probability of labor participation. Stata (version 16.0, Stata, Computer Resource Center, College Station, TX, USA) was used for data analysis.

#### 2.3.2. Years of labor participation

For converting the excess labor participation rate into excess years of labor participation, Milligan and Wise measured the delayable retirement age for middle-aged and older Americans based on mortality rates, and demonstrated that the sum of the excess labor participation rates at each age group was equal to their delayable years of retirement. Excess years of work could be obtained by multiplying the additional labor participation rate for each age group by the age interval, according to Milligan and Wise's formula (45). Therefore, this method was used to convert the excess work rate of women into years.

#### 2.3.3. Critical assumptions

Several assumptions exist in application of the CMR health measurement method. To ensure the scientific precision of the study, four items were summarized based on differences in background as follows. First, it is assumed that health status has been completely included and there are no missing or omitted health variables. With increasing age and poorer health, it is easy to overestimate the additional work capacity of older women, therefore multi-dimensional health behaviors, including smoking, are included in this paper to ensure that health status is completely assessed as much as possible. Second, it is assumed that health status has an equal motivational effect on women aged 45–49 with women aged 50 and above. Third, it is assumed that all non-labor participating women aged 45–49 are influenced by health factors to exit the labor market, and the effects of other non-health factors, such as institutional factors like pensions, are not considered. If present, they are prone to underestimate excess work capacity. Given that the highest legal retirement age for Chinese women is 55 (8), women in the 45–49 age group were selected for this study in order to avoid the influence of non-health factors as much as possible. However, it has been pointed out that early retirement is widespread in China (46), and although a sample from the younger pre-retirement group was used in this study to reduce this error, the excess work capacity obtained is likely to be underestimated due to the presence of early retirement. However, in terms of delayed retirement, this underestimation does not bring a fundamental change to the conclusions of this paper. Fourth, health and employment endogeneity issues, like the reverse effect of labor participation on health, are not considered.

## 3. Results

### 3.1. Descriptive statistics

The labor participation rates, individual characteristics, and prevalence of disease among urban and rural women by age group are described in Table 1. Labor participation rate generally decreases with age. The rate is lower in urban women than in rural women and shows a significant downward trend at the age of 55, while the rate of rural women decreases more smoothly, from 84.1% at the age of 45–49 to 42.2% at the age of 70–74, with a total decrease of 41.9%. The rate of urban women plunges to 59.7% just after 55. With regards to health, it was found that PVW health index along with extremely good and relatively good self-rated health are gradually decreased with age, while the proportion of fair, bad, and extremely bad increased, so did most of the remaining health indicators. And the health status of urban women was generally higher than rural women. However, depression trended oppositely as depression levels decreased with age, which is consistent with the observation that subjective wellbeing or psychological health usually improves at older ages. The proportion of women aged 45–54 with secondary specialized education and above is relatively high, and there is an obvious downward trend after the age of 54, with the educational level of urban women being significantly higher than that of rural women, which is consistent with different generations of Chinese. The proportion of divorce and widowhood increases as age increases, so the proportion of non-married women gradually increases.

**Table 1:** Labor participation rates, individual characteristics and prevalence of disease by age group.

		**Rural women age group**						**Urban women age group**					
		**45–49**	**50–54**	**55–59**	**60–64**	**65–69**	**70–74**	**45–49**	**50–54**	**55–59**	**60–64**	**65–69**	**70–74**
Work		0.841	0.772	0.735	0.685	0.605	0.422	0.678	0.483	0.286	0.217	0.126	0.081
PVW index		58.493	55.453	51.565	47.774	41.789	36.789	65.857	62.736	55.915	52.140	48.362	47.194
SRH	Very good	0.046	0.072	0.068	0.062	0.063	0.051	0.049	0.093	0.078	0.058	0.082	0.059
	Good	0.106	0.093	0.099	0.095	0.093	0.112	0.152	0.131	0.118	0.129	0.075	0.121
	Fair	0.543	0.498	0.484	0.465	0.437	0.413	0.582	0.577	0.568	0.556	0.579	0.562
	Poor	0.269	0.292	0.305	0.327	0.353	0.367	0.203	0.173	0.203	0.229	0.222	0.230
	Very poor	0.036	0.045	0.044	0.052	0.054	0.057	0.014	0.026	0.033	0.029	0.042	0.028
CESDscore		9.656	10.080	10.250	10.750	10.850	10.810	8.125	7.847	8.156	8.263	8.121	7.684
Physical limits	one	0.144	0.142	0.176	0.192	0.205	0.216	0.089	0.094	0.130	0.162	0.171	0.174
	Many	0.060	0.089	0.120	0.147	0.230	0.309	0.027	0.039	0.078	0.088	0.121	0.135
ADL: Any		0.029	0.033	0.045	0.071	0.092	0.132	0.014	0.016	0.041	0.036	0.070	0.068
IADL: Any		0.103	0.123	0.155	0.195	0.237	0.274	0.043	0.059	0.094	0.104	0.139	0.130
Chronicdisease	One	0.297	0.287	0.294	0.286	0.259	0.242	0.330	0.286	0.266	0.230	0.219	0.187
	Many	0.372	0.426	0.472	0.519	0.569	0.580	0.379	0.445	0.524	0.614	0.674	0.668
Disability		0.022	0.035	0.036	0.043	0.057	0.057	0.030	0.036	0.027	0.037	0.041	0.037
Psychiatric condition		0.031	0.032	0.034	0.044	0.052	0.051	0.019	0.030	0.029	0.040	0.048	0.025
Eyesight poor		0.046	0.059	0.060	0.085	0.123	0.138	0.032	0.039	0.054	0.077	0.088	0.064
Hearing poor		0.051	0.069	0.072	0.098	0.125	0.173	0.049	0.053	0.068	0.081	0.112	0.121
Falldown		0.162	0.174	0.197	0.236	0.253	0.276	0.167	0.174	0.220	0.192	0.224	0.214
Fracture		0.008	0.010	0.015	0.017	0.017	0.024	0.009	0.009	0.014	0.005	0.013	0.018
Smoke		0.056	0.063	0.073	0.106	0.109	0.141	0.035	0.065	0.049	0.076	0.066	0.126
Drink		0.075	0.075	0.076	0.074	0.099	0.087	0.083	0.077	0.079	0.069	0.077	0.066
Married		0.970	0.949	0.914	0.871	0.782	0.641	0.937	0.926	0.883	0.847	0.795	0.607
Education:	Illiteracy	0.214	0.273	0.444	0.518	0.514	0.615	0.037	0.059	0.113	0.158	0.203	0.195
	Elementary	0.515	0.426	0.369	0.399	0.431	0.333	0.238	0.236	0.261	0.367	0.402	0.434
	Middle and Above	0.271	0.301	0.187	0.082	0.054	0.052	0.726	0.705	0.627	0.476	0.395	0.372
Health Insurance		0.921	0.908	0.926	0.915	0.922	0.900	0.896	0.919	0.916	0.907	0.945	0.916
Observation		4,003	4,716	4,772	4,975	3,753	3,448	791	1,085	1,209	1,211	997	977

### 3.2. Regression analysis of women's health and labor participation

#### 3.2.1. Regression analysis of women's health and labor participation: Women aged 45–74

Considering that women aged 45–49 have not reached retirement age and may not reflect the association between health and labor participation well, a regression was first conducted for all women aged 45–74. Table 2 demonstrates the results of the Probit regression between labor participation and health for women aged 45–74 based on all health variables in Model 1. The majority of health indicators were associated with labor participation. Worse health, one or more physical limits, numbers of ADL or IADL limitations equal to or above 1, multiple chronic conditions, disabilities, psychiatric conditions, and smoking were all associated with lower labor participation. However, CESD score, number of falls, and the consumption of alcohol were positively associated with labor participation, which could be due to a multiple covariance phenomenon caused by entering excessive health indicators simultaneously. To avoid such problems, health indicators were excluded in the baseline regressions of Table 3, which used the PVW index instead of a series of health indicators. Women who married, with rural residence, and with health insurance have higher labor participation, but the phenomenon worth noting is that highly educated women are associated with lower labor participation.

**Table 2:** Regression of women's health and labor participation (*N* = 31,937).

**Variable**		**45–74 women**	
		**Coef**.	**S.E**.
SRH	Good	0.1775^***^	(0.0387)
	Fair	0.0671^*^	(0.0325)
	Poor	−0.0439	(0.0352)
	Very poor	−0.1989^***^	(0.0493)
CESDscore		0.0173^***^	(0.0013)
Physical limits	One	−0.1802^***^	(0.0209)
	Two and more	−0.5819^***^	(0.0252)
ADL	Yes	−0.3319^***^	(0.0351)
IADL	Yes	−0.1987^***^	(0.0231)
Chronicdisease	One	−0.0418	(0.0220)
	Two and more	−0.2541^***^	(0.0206)
Disability	Yes	−0.1149^**^	(0.0400)
Psychiatric condition	Yes	−0.1488^***^	(0.0402)
Eyesight poor	Yes	−0.0212	(0.0292)
Hearing poor	Yes	0.0116	(0.0271)
Falldown	Yes	0.1197^***^	(0.0192)
Fracture	Yes	−0.1345^*^	(0.0635)
Smoke	Yes	−0.2161^***^	(0.0270)
Drink	Yes	0.3784^***^	(0.0299)
Married	Yes	0.4327^***^	(0.0217)
Education	Elementary	−0.0087	(0.0177)
Middle school and above	−0.0249^***^	(0.0221)
Health Insurance	Yes	0.1333^***^	(0.0270)
Residence	Rural	1.0970^***^	(0.0209)

* p < 0.05,

** p < 0.01,

*** p < 0.001.

**Table 3:** Benchmark regression of women's health and labor participation (PVW Index).

**Variable**	**45–49**	**Rural women age group**					
	** All women**	**45–49**	**50–54**	**55–59**	**60–64**	**65–69**	**70–74**
	**A**	**B**	**C**	**D**	**E**	**F**	**G**
PVW Index	0.0088^***^	0.0078^***^	0.0069^***^	0.0068^***^	0.0074^***^	0.0092^***^	0.0091^***^
	(0.0008)	(0.0009)	(0.0007)	(0.0007)	(0.0007)	(0.0007)	(0.0007)
**Married**							
Yes	−0.0441	−0.031	0.2344^**^	−0.0326	0.1383*	0.3768^***^	0.2571^***^
	(0.1120)	(0.1378)	(0.0880)	(0.0715)	(0.0550)	(0.0507)	(0.0459)
**Education**							
Elementary	0.1458^*^	0.0959	−0.077	−0.1449^**^	−0.1405^***^	−0.0643	−0.0357
	(0.0590)	(0.0611)	(0.0507)	(0.0443)	(0.0395)	(0.0437)	(0.0469)
Middle school and above	0.0759	0.0621	−0.1872^***^	−0.2834^***^	−0.2660^***^	−0.4937^***^	−0.3993^***^
	(0.0650)	(0.0698)	(0.0544)	(0.0538)	(0.0690)	(0.0949)	(0.1037)
**Health Insurance**				
Yes	0.3397^***^	0.3709^***^	0.0571	0.4426^***^	0.0258	0.1875^*^	0.0722
	(0.0718)	(0.0818)	(0.0706)	(0.0709)	(0.0665)	(0.0785)	(0.0738)
**Residence**				
Rural	0.6094^***^	–	–	–	–	–	–
	(0.0580)	–	–	–	–	–	–
Observations	4,794	4,003	4,716	4,772	4,975	3,753	3,448

Standard errors in parentheses,

* p < 0.05,

** p < 0.01,

*** p < 0.001.

#### 3.2.2. Benchmark regression: Women aged 45–49

In this study, three benchmark groups were introduced, the first one utilizes the Cutler traditional approach of including all women aged 45–49 as a benchmark. Column A in Table 3 demonstrates the relationships between labor participation and health among all women aged 45–49, indicating a positive relationship between health indicators and labor participation. Marriage and educational status were not statistically significant stratifiers in the younger age group.

#### 3.2.3. Benchmark regression: Rural women aged 45–49

The second benchmark group of this paper is rural women aged 45–49, which is used to predict the excess work capacity of urban women. Column B of Table 3 reports the health and labor participation regression results of rural women aged 45–49. As can be seen, the PVW coefficient of 0.0078 is lower than that of all women in the 45–49 age group. The PVW index is a 0–100 percentile, with larger representing better health, so the regression coefficient of the PVW index for rural women aged 45–49 is lower than that of all women in the same age group, implying that rural women Chinese rural women's work decisions are less responsive to health levels. As expected, rural women have a higher labor participation rate, but their health level is worse, and therefore have lower PVW coefficients.

#### 3.2.4. Benchmark regression: Rural women by age group

The previous two benchmark groups used young cohorts. It is expected that, although the results can be more sensitive when using young cohorts as the benchmark, it is more likely to exaggerate capacity to work than when using women of the same age group as the benchmark. Columns C-G in Table 3 demonstrate the relationship between labor participation and health among every age group of rural women. Horizontally, PVW coefficients of rural women aged 45–59 gradually decreased, indicating that as rural women grew older, declining health had no significant impact on labor force participation, which proves that rural women tend to continue working as long as their health allows. The PVW coefficients of rural women aged 60–74 gradually increased, indicating that as rural women age further, and the relationship between labor force participation and health status becomes more sensitive. Longitudinally, marriage and education level are statistically significant stratifiers in rural women of higher age groups. Unmarried or highly educated women are often associated with lower labor participation, so it is necessary to conduct further analysis of educational heterogeneity.

### 3.3. Measurement of excess labor participation rate

#### 3.3.1. All (urban and rural) women: Benchmark of women aged 45–49

The results of the two-model measures of predicted work capacity and delayable work capacity for women in each age group were summarized in Table 4. Based on the regression results of the women aged 45–49 in Table 3, further predictions were conducted for women aged 50–74, on the subsequent measure of excess work capacity. The results of model 1 are still given in order to compare the two models. Actual labor participation rates of women aged 50–74 were high in all age groups, with 34.64% still working in women aged 70–74, however, the actual labor participation rate of urban women aged 70–74 is only 8.09%. As rural women account for nearly 80% of this study, the larger sample resulted in a higher labor participation rate for all women and a lower ability to excess work. Therefore, further measurements were conducted to stratify urban and rural women. It was found in model 1 that the excess labor participation rates of rural women aged 50–54, 55–59, 60–64, 65–69, and 70–74 were 4.34, 7.19, 11.12, 17.98, and 34.61%, respectively, while the rates of urban women in the same age group were 30.69, 50.19, 56.55, 64.50, and 67.19%, respectively, about 2–3 times higher than those of rural women.

**Table 4:** Calculation of excess work capacity (%): all 45–49 women as benchmark.

	**Age group**	**Obs**	**Actual working**	**Model 1: All health variables**		**Model 2: PVW index**	
				**Predicted working**	**Excess working capacity**	**Predicted working**	**Excess working capacity**
All	50–54	5,801	71.82	78.47	6.65	78.69	6.87
	55–59	5,981	64.44	76.86	12.42	77.43	12.99
	60–64	6,186	59.36	75.84	16.48	76.58	17.22
	65–69	4,750	50.44	74.08	23.64	75.28	24.84
	70–74	4,425	34.64	71.70	37.06	73.86	39.22
Urban	50–54	1,085	45.70	78.21	32.51	78.98	33.28
	55–59	1,209	25.90	77.36	51.46	78.81	52.91
	60–64	1,211	19.50	77.77	58.27	78.27	58.77
	65–69	997	13.14	77.65	64.51	77.14	64.00
	70–74	977	8.33	77.60	69.27	75.28	66.95
Rural	50–54	4,716	77.23	81.41	4.18	81.57	4.34
	55–59	4,772	73.51	80.20	6.69	80.70	7.19
	60–64	4,975	68.52	79.04	10.52	79.64	11.12
	65–69	3,753	60.48	77.47	16.99	78.46	17.98
	70–74	3,448	42.17	74.86	32.69	76.78	34.61

#### 3.3.2. Urban women: Rural women as benchmark

The predicted results based on rural women aged 45–49 are displayed in Table 5, and the excess work capacity predicted based on rural women aged 45–49 is higher compared to that based on all women aged 45–49. The capacity of model 1 shows an increase from 32.51 to 36.94%. The capacity increases from 55.46 to 55.05% for urban women aged 50–54, and the capacity of model 2 is higher than that of model 1 by about 3% for all age groups.

**Table 5:** Calculation of excess work capacity (%): different benchmark.

**Age group**	**Obs**	**Actual working**	**Model 1: All health variables**		**Model 2: PVW index**	
			**Predicted working**	**Excess working capacity**	**Predicted working**	**Excess working capacity**
50–54	1,085	45.70	82.64	36.94	83.11	37.41
55–59	1,209	25.90	80.95	55.05	82.15	56.25
60–64	1,211	19.50	80.28	60.78	81.82	62.32
65–69	997	13.14	79.03	65.89	81.66	68.52
70–74	977	8.33	78.34	70.01	81.77	73.44
50–54	1,085	45.70	75.49	29.79	75.8	30.10
55–59	1,209	25.90	70.72	44.82	71.35	45.45
60–64	1,211	19.50	65.45	45.95	65.82	46.32
65–69	997	13.14	54.81	41.67	55.82	42.68
70–74	977	8.33	38.81	30.48	39.36	31.03

#### 3.3.3. Urban women: Rural women of different age groups as benchmarks

Benchmarking exclusively on high labor participation rates of rural women aged 45–49 may overestimate the capacity of urban women. The second part of Table 5 compares the capacity of rural women with urban women at the same age and finds more conservative results for urban women, whose excess work capacity no longer increases with age, but decreases after reaching a peak of 45.95% at age 60–64.

### 3.4. Heterogeneity analysis of educational level

Delayable labor participation rates among women of different educational levels by age group are reported in Table 6. The largest capacity was obtained from the benchmark group of rural women aged 45–49, and the most conservative result was obtained from the benchmark group of illiterate women of all ages. In terms of the actual participation rate, women with higher educational levels tend to have lower labor participation rates, with the labor participation rates of women aged 50–54, 55–59, 60–64, 65–69, and 70–74 in secondary specialized education and above being 64.27, 48.65, 33.85, 20.53, and 12.57%, respectively. The participation rate of illiterate women aged 70–74 was at 41.82%. As for estimated labor participation rates, differences among women with different educational levels were not significant, all around 60–80%. However, when comparing excess work capacity, illiterate women tend to be lower, while women with secondary specialized education and above tend to increase more distinctly with age. The excess labor participation rates calculated from all women, urban and rural women, and women with different educational levels, show the results of the PVW score model are similar with those of the full health variable model, indicating good statistical stability.

**Table 6:** Excess work capacity of women with different education levels (%).

**Age group**	**Education**	**Obs**	**Actual working**	**Benchmark: Women 45–49**		**Benchmark: Rural women 45–49**		**Benchmark: By education**	
				**Predicted working**	**Excess working capacity**	**Predicted working**	**Excess working capacity**	**Predicted working**	**Excess working capacity**
**50–54**									
	Illiteracy	1,351	77.94	80.26	2.32	80.98	3.04	78.16	0.22
	Elementary	2,267	74.06	79.47	5.41	82.80	8.74	77.35	3.29
Middle school and above		2,183	65.69	77.46	11.77	82.31	16.62	75.62	9.93
**55–59**									
	Illiteracy	2,256	74.16	79.38	5.22	80.61	6.45	77.37	3.21
	Elementary	2,074	66.68	78.14	11.46	82.40	15.72	75.94	9.26
Middle school and above		1,651	48.33	74.59	26.26	81.76	33.43	72.48	24.15
**60–64**									
	Illiteracy	2,769	68.51	77.82	9.31	79.62	11.11	75.68	7.17
	Elementary	2,431	58.86	77.07	18.21	82.00	23.14	74.81	15.95
Middle school and above		986	34.89	71.80	36.91	81.39	46.50	69.48	34.59
**65–69**									
	Illiteracy	2,133	58.23	76.53	18.30	79.31	21.08	75.00	16.77
	Elementary	2,019	50.72	75.29	24.57	81.21	30.49	73.30	22.58
Middle school and above		598	21.74	69.07	47.33	80.86	59.12	66.87	45.13
**70–74**									
	Illiteracy	2,312	40.53	75.51	34.98	78.57	38.04	74.42	33.89
	Elementary	1,572	33.59	73.41	39.82	80.76	47.17	71.84	38.25
Middle school and above		541	12.57	68.62	56.05	80.24	67.67	66.38	53.81

### 3.5. Measurement of excess work capacity years

Table 7 reports the measurement results of the PVW index model of women's excess working years. When urban and rural areas are not stratified, it is found that the excess working capacity of all women aged 50–64 is only 2 years, and that of all women aged 50–74 is 5.1 years, therefore excess working capacity is not obvious. It may be that there are more rural women in the study, which leads to an increase of the “endless labor” sample, so the predicted excess working capacity is small. Subsequently, urban and rural women were calculated separately, and it can be seen that when taking all women from 45 to 49 as the benchmark, the excess working years in urban and rural areas from 50 to 64 are 7.2 and 1.1 years, respectively, and the excess working years in age group from 50 to 74 are 13.8 and 3.8 years, respectively. The excess working capacity of urban women is huge. Therefore, to further measure the excess working ability of urban women, different rural women groups were taken as the benchmark group. When using rural women aged 45–49 as the benchmark, the excess working ability of urban women aged 50–64 and 50–74 is 7.8 and 14.9 years, respectively. This was done to avoid overestimating the excess working ability of urban women. As expected, the benchmark for rural women in the same age group gives a more conservative result of 6.1 and 9.8 years of extra work for urban women aged 50–64 and 50–74, respectively.

**Table 7:** Years of excess work for women (Years).

	**All**	**Residence**		**Education**		
		**Urban**	**Rural**	**Illiteracy**	**Elementary**	**Middle school and above**
**Benchmark: Women 45–49**						
50–64	1.9	7.2	1.1	0.8	1.8	3.7
50–74	5.1	13.8	3.8	3.5	5.0	8.9
**Benchmark: Rural women 45–49**						
50–64	–	7.8	–	1.0	2.4	4.8
50–74	–	14.9	–	4.0	6.3	11.2
**Benchmark: Age specific rural women age group**						
50–64	–	6.1	–	–	–	–
50–74	–	9.8	–	–	–	–
**Benchmark: By education**						
50–64	–	–	–	0.5	1.4	3.4
50–74	–	–	–	3.1	4.5	8.4

When measured separately in terms of educational level, it was found that the excess years of work were increased with increasing educational level. The increase was 3.5–4.8 years for women aged 50–64 with middle school and above, and 8.4–11.2 years for women aged 50–74, which illustrates that older women with higher educational level tend to possess higher work potential.

## 4. Discussion

The current policy reform on delayed retirement in China has not yet been implemented, and there are few studies on excess work capacity based on health perspective in China. The excess work capacity of Chinese women was estimated using a Probit model by comprehensively assessing women's health status using a variety of single health variables and PVW health coefficients. There is one study closely related by Cutler et al. who estimated the excess work capacity of the older workforce using data from 12 countries participating in the *International Social Security Project* (ISS), including the UK, Japan, Germany, and Italy, which conducted an international comparison (40). Unlike previous studies, this paper includes two new benchmark groups, young rural women in China and rural women in the same age group. Compared to Cutler et al. who took a single young cohort as the baseline group for their study, China, a developing country, has a special study context with urban-rural background differences and rural women with endless labor behavior (13, 47, 48). Rural women in China tend not to have a clear concept of retirement and will work if their health allows. Therefore, the use of the same age group of rural women as the benchmark group, ensures more accurate results than using a younger cohort and is less likely to have results that exaggerate the excess work capacity of older adults.

Health level is associated with labor force participation among older adults. Older women with better health tend to remain in the labor force, which is consistent with previous research (49, 50). The effect of health on labor participation is not only manifested in self-rated health, but objective health measures such as chronic illness, ADL and IADL are significantly associated with labor participation (27). It is important to note that Chinese rural women generally have lower health levels than urban women. Previous studies have also demonstrated that rural Chinese women are far less susceptible to retirement due to health issues than urban women (47). In other words, rural women are more likely to continue working as long as their health allows until their health deteriorates (15). This phenomenon is common in China, but this idea often leads rural women with health problems to remain in the labor market and thus experience health deterioration, exacerbating urban-rural health inequalities (51). Given the gap between rural and urban areas, the government should provide appropriate assistance to middle-aged and older women who continue to work in agriculture due to economic factors and improve social welfare policies for middle-aged and older rural women to narrow the health gap between urban and rural women. The retirement age should also be set to maintain proper consideration of women's health levels and implement a flexible retirement policy (52).

The results of this study also suggest there is an urban-rural heterogeneity in the excess work capacity of older women. Urban women have significantly higher excess capacity to work than rural women, but urban women with better health have much lower actual labor participation rates than rural women. This suggests that urban women have health levels to support delayed retirement, but it does not mean that urban women are willing to continue working after retirement or support delayed retirement policies. Many scholars have investigated women's willingness to delay retirement and found that most urban women workers oppose delaying retirement (53). It is mainly due to two reasons, one is that urban women have better social security resources, they can get access to better social security services and benefits (54). In addition, the three-child policy is currently being advocated in China (55), due to China's strong traditional intergenerational ties and the inadequacy of the current childcare system (56, 57), more and more women prefer to retire to care for their grandchildren to reduce the pressure on their children (58, 59). In the absence of social support, many women are physically and psychologically exhausted by family (60). Therefore, in the formulation of delayed retirement policies, urban-rural differences should be considered according to health status. For urban women, a corresponding retirement incentive should be established to encourage women in better health to remain in the labor market and provide certain subsidies (61), while supporting policies such as infant and childcare services should also be implemented one after another. The public welfare services for childcare to some extent can weaken the grandparenting responsibility and intensity of the older women and in turn can enable them to remain in the labor market and prolong their working life (62).

Finally, educational heterogeneity was found in the excess work capacity of older women. Excess work capacity increases with education and the predicted working rate is higher for highly educated women, but the actual labor participation rate is lower. However, in developed countries such as Denmark, the extra work capacity of highly educated women is instead lower than that of less educated women (63). This shows there is currently a huge labor potential for highly educated older women of China. Along with Chinese women's education levels significantly rising (43), women's initial age of entry into the labor market has been delayed, while the retirement age has remained the same implying that women are working fewer years (64). Furthermore, women with higher education are generally in better health, especially the older people (65), thus, women with more education are more capable of delaying retirement (66). Therefore, considering the characteristics of knowledgeable women with long years of education, high starting age for employment, abundant human capital stock, and generally better health, it is possible to take the lead in implementing a delayed retirement policy in medical, scientific research, and higher education sectors where knowledgeable women are concentrated (67), so as to make reasonable use of human resources of highly educated female and gradually narrow the retirement age gap between males and women (52).

## 5. Limitations

This paper also has some limitations. First, this study is based on survey data from women aged 45 and above, which, although relatively representative, is the time women start to think about retirement and is not representative of the entire female population. Secondly, it is undeniable that the estimation method in this paper relies on many assumptions. For example, the endogeneity of labor force participation and health levels are not considered. Although multiple benchmark groups and different health measures were used to ensure the robustness of the findings as much as possible, given the importance of delayed retirement reform, we argue that more predictive research on the additional work capacity of the older population is still needed. Third, the delayed retirement age policy targets full-time workers, and the labor force participation behavior in this study is examined for both full-time and part-time women, and we hope that further distinctions can be made in future studies to better promote policy improvement.

## 6. Conclusion

China has announced a delayed retirement system, but the specific delayed retirement policy still needs to be formulated (8). As health is an essential factor affecting the employment of the older people (22), few studies in China have been conducted to explain whether the older population has sufficient health capacity to cooperate with implementing the delayed retirement policy from a health perspective. This study finds that older women in China still have some excess working capacity and room for delayed retirement based on women's health perspective, which further enriches the measurement of retirement age and provides some theoretical support for the delayed retirement policy. As the health level of older women continues to improve, if the labor potential of older women can be further explored, it will help further mitigate the adverse effects of population aging and realize the positive aging strategy (52). However, the implications of delaying retirement age reform are broad and far-reaching. In the future, there is still a need to use more detailed information on employment and health, taking into account a variety of integrated factors including health levels. A more in-depth exploration of the gender differences that exist in the retirement ages of men and women in China is needed to facilitate large-scale delayed retirement policy reform.

## Data availability statement

The datasets presented in this study can be found in online repositories. The names of the repository/repositories and accession number(s) can be found in the article/Supplementary material.

## Ethics statement

The studies involving human participants were reviewed and approved by Biomedical Ethics Review Committee of Peking University. The patients/participants provided their written informed consent to participate in this study.

## Author contributions

YZ and YF conceived and designed the study and supervised the data analysis. XC and YZ wrote the paper. XC performed all statistical analyses. YZ and XC contributed to revising the paper. All authors have read and agreed to the published version of the manuscript.

## References

[1] Etgeton S. The effect of pension reforms on old-age income inequality. Labour Econ. (2018) 53:146–61. 10.1016/j.labeco.2018.05.006

[2] Basten S Jiang Q. China's family planning policies: recent reforms and future prospects. Stud Fam Plann. (2014) 45:493–509. 10.1111/j.1728-4465.2014.00003.x 25469931

[3] Kim KW Kim OS. Super aging in South Korea unstoppable but mitigatable: a sub-national scale population projection for best policy planning. Spat Demogr. (2020) 8:155–73. 10.1007/s40980-020-00061-8 34222615 PMC8248505

[4] Chen R Xu P Song P Wang M He J. China has faster pace than Japan in population aging in next 25 years. BioSci Trends. (2019) 13:287–91. 10.5582/bst.2019.01213 31434814

[5] Liao P Su H Pamucar D. Will ending the one-child policy and raising the retirement age enhance the sustainability of China's basic pension system? Sustainability. (2020) 12:8172. 10.3390/su12198172

[6] Ren X Xi H Zhai S Zhou M. Research on the accumulation effect of pension income and payments caused by progressive retirement age post-ponement policy in China. J Aging Soc Policy. (2019) 31:155–69. 10.1080/08959420.2018.1500859 30358511

[7] Hu H Wang W Feng D Yang H. Relationships between migration and the fiscal sustainability of the pension system in China. PLoS ONE. (2021) 16:e0248138. 10.1371/journal.pone.0248138 33690624 PMC7946295

[8] Feng Q Yeung W-JJ Wang Z Zeng Y. Age of retirement and human capital in an aging China, 2015–2050. Eur J Popul. (2018) 35:29–62. 10.1007/s10680-018-9467-3 30976267 PMC6357252

[9] QinBo G Han D. Disscuss on women's statutory pensionable age in China. Collect Women's Stud. (2009) 32–37. Available online at: https://oversea.cnki.net/KCMS/detail/detail.aspx?dbcode=CJFD&dbname=CJFD2009&filename=FNYJ200906006&uniplatform=OVERSEA&v=3BgfGn2nHh5hfz6LCuqwcGBRpiK0LYl45xdmM5TJqu6M0YDTGu_LF6-kboEuJqsv

[10] OECD. Pensions at a Glance 2015: OECD and G20 indicators. OECD (2015).

[11] OECD. Working Better With Age. OECD (2019).

[12] Elder TE. The predictive validity of subjective mortality expectations: evidence from the health and retirement study. Demography. (2013) 50:569–89. 10.1007/s13524-012-0164-2 23154962

[13] Giles J Lei X Wang G Wang Y Zhao Y. One country, two systems: evidence on retirement patterns in China. J Pension Econ Finance. (2021) 1–23. 10.1017/S1474747221000391 PMC10187591 37197054

[14] Wu J. Why is the policy of delayed retirement “delayed”?—An analysis of policy agenda in the multiple-streams framework. J Southwest Univ. (2021) 47:59–70. 10.13718/j.cnki.xdsk.2021.03.006

[15] Mountian AG Montoya Diaz MD. Effects of retirement on the health of elderly people in São Paulo, Brazil. Appl Econ. (2020) 52:2991–3003. 10.1080/00036846.2019.1697797

[16] Boissonneault M de Beer J. Assessing the capacity to work among older workers: a survival analysis of retirement behavior. Work Aging Retirement. (2022) 8:38–50. 10.1093/workar/waab008

[17] Barslund M Bauknecht J Cebulla A. Working conditions and retirement: How important are HR policies in prolonging working life? MREV. (2019) 30:120–41. 10.5771/0935-9915-2019-1-120

[18] van Rijn RM Robroek SJW Brouwer S Burdorf A. Influence of poor health on exit from paid employment: a systematic review. Occup Environ Med. (2014) 71:295–301. 10.1136/oemed-2013-101591 24169931

[19] Bloemen HG. The effect of private wealth on the retirement rate: an empirical analysis. Economica. (2011) 78:637–55. 10.1111/j.1468-0335.2010.00845.x

[20] Giesecke M. The effect of benefit reductions on the retirement age: the heterogeneous response of manual and non-manual workers. Rev Income Wealth. (2018) 64:213–38. 10.1111/roiw.12257

[21] Roberts J Rice N Jones AM. Early retirement among men in Britain and Germany: how important is health? Geneva Pap Risk Insur Issues Pract. (2010) 35:644–67. 10.1057/gpp.2010.24

[22] McGarry K. Health and retirement: do changes in health affect retirement expectations? J Hum Resour. (2004) 39:624. 10.2307/355899031011107

[23] Boskin MJ Hurd MD. The effect of social security on early retirement. J Public Econ. (1978) 10:361–77. 10.1016/0047-2727(78)90052-X

[24] Haveman R Wolfe B Kreider B Stone M. Market work, wages, and men's health. J Health Econ. (1994) 13:163–82. 10.1016/0167-6296(94)90022-1 10138024

[25] Bound J. Self-Reported vs. Objective Measures of Health in Retirement Models. Cambridge, MA: National Bureau of Economic Research (1989). 10.3386/w2997

[26] Anderson KH Burkhauser RV. The importance of the measure of health in empirical estimates of the labor supply of older men. Econ Lett. (1984) 16:375–80. 10.1016/0165-1765(84)90192-7

[27] Datta Gupta N Larsen M. The impact of health on individual retirement plans: self-reported vs. diagnostic measures. Health Econ. (2010) 19:792–813. 10.1002/hec.1523 19582695

[28] Pienta AM Hayward MD. Who expects to continue working after age 62? The retirement plans of couples. J Gerontol B Psychol Sci Soc Sci. (2002) 57:S199–208. 10.1093/geronb/57.4.S199 12084790

[29] French E Jones JB. Health, health insurance, and retirement: a survey. Ann Rev Econ. (2017) 29:383–409. 10.1146/annurev-economics-063016-10361611010239

[30] Currie J Madrian BC. Chapter 50 Health, health insurance and the labor market., Handbook of Labor Economics. Elsevier (1999). p. 3309–3416 10.1016/S1573-4463(99)30041-9

[31] Kapteyn A Smith JP van Soest A. Dynamics of work disability and pain. J Health Econ. (2008) 27:496–509. 10.1016/j.jhealeco.2007.05.002 18180063 PMC3654673

[32] Stern S. Measuring the effect of disability on labor force participation. J Hum Resour. (1989) 24:361–95. 10.2307/14581934706829

[33] Poterba J Venti S Wise D. The Asset Cost of Poor Health. Cambridge, MA: National Bureau of Economic Research (2010). 10.3386/w16389 PMC5495653 21604611

[34] Poterba J Venti S Wise DA. Health, education, and the postretirement evolution of household assets. J Hum Cap. (2013) 7:297–339. 10.1086/673207 24904710 PMC4043284

[35] Milligan K Schirle T. Health and capacity to work of older Canadians: gender and regional dimensions. Canad Public Policy. (2018) 44:159–72. 10.3138/cpp.2017-028

[36] Bingley P Gupta ND Pedersen P. Health Capacity to Work at Older Ages in Denmark. Cambridge, MA: National Bureau of Economic Research (2016). 10.3386/w22018

[37] Usui E Shimizutani S Oshio T. Health Capacity to Work at Older Ages: Evidence from Japan. Cambridge, MA: National Bureau of Economic Research (2016). 10.3386/w21971

[38] García-Gómez P Jimenez-Martin S Castelló JV. Health Capacity to Work at Older Ages: Evidence from Spain. Cambridge, MA: National Bureau of Economic Research (2016). 10.3386/w21973

[39] Wise DA. Facilitating longer working lives: international evidence on why and how. Demography. (2010) 47:S131–49. 10.1353/dem.2010.0000 21302423 PMC5870615

[40] Cutler DM Meara E Richards-Shubik S. Health and work capacity of older adults: estimates and implications for social security policy. SSRN J. (2013). 10.2139/ssrn.2577858

[41] Oshio T Shimizutani S. Health capacity to work and its long-term trend among the Japanese elderly. J Jpn Int Econ. (2019) 51:76–86. 10.1016/j.jjie.2018.12.001

[42] Zhao Y Hu Y Smith JP Strauss J Yang G. Cohort Profile: The China health and retirement longitudinal study (CHARLS). Int J Epidemiol. (2014) 43:61–8. 10.1093/ije/dys203 23243115 PMC3937970

[43] Du P Li L. The development trend of education achievement of the elderly in China. Populat Develop. (2022) 28:59–67. Available online at: https://oversea.cnki.net/KCMS/detail/detail.aspx?dbcode=CJFD&dbname=CJFDLAST2022&filename=SCRK202201006&uniplatform=OVERSEA&v=5OKka5ltV4VpzNaGf8Z34vZc8TGPadopmmnxl37_K2xEhTLAqG_MdI1wFWeVqPX933143189

[44] Silverstein M Cong Z Li S. Intergenerational transfers and living arrangements of older people in rural China: consequences for psychological wellbeing. J Gerontol B Psychol Sci Soc Sci. (2006) 61:S256–66. 10.1093/geronb/61.5.S256 16960239

[45] Coile C Milligan K Wise D. Health Capacity to Work at Older Ages: Evidence from the U.S. Cambridge, MA: National Bureau of Economic Research (2016). 10.3386/w21940

[46] Feng J Hu Y. An empirical study on early retirement in urban China. Chinese J Popul Sci. (2008) 88–94. Available online at: https://oversea.cnki.net/KCMS/detail/detail.aspx?dbcode=CJFD&dbname=CJFD2008&filename=ZKRK200804013&uniplatform=OVERSEA&v=s2LpIWrI6i8losNIRkoz7JAeDi8m61g_LRiB-6-LTt9DQQwMWvouHMVvT5je_unv

[47] Yu M-Y Sarri R. Women's health status and gender inequality in China. Soc Sci Med. (1997) 45:1885–98. 10.1016/S0277-9536(97)00127-5 9447637

[48] Mitra S Gao Q Chen W Zhang Y. Health, work, and income among middle-aged and older adults: a panel analysis for China. J Econ Ageing. (2020) 17:100255. 10.1016/j.jeoa.2020.10025533602174

[49] Dwyer DS Mitchell OS. Health problems as determinants of retirement: are self-rated measures endogenous? J Health Econ. (1999) 18:173–93. 10.1016/S0167-6296(98)00034-4 10346352

[50] Orszag JM Fabel O. The economics of pensions and variable retirement schemes. Economica. (1995) 62:411. 10.2307/2554875

[51] Glauber R. Rural depopulation and the rural-urban gap in cognitive functioning among older adults. J Rural Health. (2022) 38:696–704 10.1111/jrh.12650 35257439 PMC10268026

[52] Liu Y Yang M Zheng H Jiang Y Gu D. Modelling a flexible retirement age to narrow pension gap: the case of China. Singapore Econ Rev. (2021) 66:1665–85. 10.1142/S0217590818420079

[53] Wang J Li X. A study of the urban professional females' attitudes toward the policy of delayed retirement. South China Populat. (2019) 34:15–23. Available online at: https://oversea.cnki.net/KCMS/detail/detail.aspx?dbcode=CJFD&dbname=CJFDLAST2019&filename=LFRK201905002&uniplatform=OVERSEA&v=7jpVpDUb690WsC9Dx4oX7NUyR0hxMPPyACB_uGLZcBHFUSVjHeoYW20GkprIhkHx

[54] Liu T Sun L. Pension reform in China. J Aging Soc Policy. (2016) 28:15–28. 10.1080/08959420.2016.1111725 26549002

[55] Kang L Jing W Liu J Ma Q Zhang S Liu M. The prevalence of barriers to rearing children aged 0-3 years following China's new three-child policy: a national cross-sectional study. BMC Public Health. (2022) 22:489. 10.1186/s12889-022-12880-z 35279114 PMC8917473

[56] Fu L Wang Y He L. Factors associated with the psychological health of caregiving older parents and support from their grown children: results from the China health and retirement longitudinal study. Int J Environ Res Public Health. (2020) 17:556. 10.3390/ijerph17020556 31952302 PMC7013421

[57] Chen F Liu G. The health implications of grandparents caring for grandchildren in China. J Gerontol B Psychol Sci Soc Sci. (2012) 67B:99–112. 10.1093/geronb/gbr132 22156630 PMC3267025

[58] Xu H. Physical and mental health of chinese grandparents caring for grandchildren and great-grandparents. Soc Sci Med. (2019) 229:106–16. 10.1016/j.socscimed.2018.05.047 29866373 PMC6261790

[59] Yang Y Meng Y Dong P. Health, security and participation: a structural relationship modeling among the three pillars of active ageing in China. Int J Environ Res Public Health. (2020) 17:7255. 10.3390/ijerph17197255 33020396 PMC7579513

[60] Chen H Hagedorn AT Peng X. Mental health resilience in older Chinese women. Innov Aging. (2017) 1:396–7. 10.1093/geroni/igx004.1433

[61] Pit SW Byles J. The association of health and employment in mature women: a longitudinal study. J Womens Health. (2012) 21:273–80. 10.1089/jwh.2011.2872 22060315 PMC3298669

[62] Van Bavel J De Winter T. Becoming a grandparent and early retirement in Europe. Eur Sociol Rev. (2013) 29:1295–308. 10.1093/esr/jct005

[63] Bingley P Gupta ND Pedersen P. Health Capacity to Work at Older Ages in Denmark. p. 34.

[64] Strulik H Werner K. 50 is the new 30—long-run trends of schooling and retirement explained by human aging. J Econ Growth. (2016) 21:165–87. 10.1007/s10887-015-9124-1

[65] Ross CE Mirowsky J. The interaction of personal and parental education on health. Soc Sci Med. (2011) 72:591–9. 10.1016/j.socscimed.2010.11.028 21227556 PMC3049298

[66] De Breij S Qvist JY Holman D Mäcken J Seitsamo J Huisman M. Educational inequalities in health after work exit: the role of work characteristics. BMC Public Health. (2019) 19:1515. 10.1186/s12889-019-7872-0 31718592 PMC6852931

[67] Zhang C Li Q Wei Y Hu Z. How long can the elderly work? A study on the additional work capacity of China's retired population studies in labor. Economics. (2020) 8:7–29. Available online at: https://oversea.cnki.net/KCMS/detail/detail.aspx?dbcode=CJFD&dbname=CJFDLAST2021&filename=LDJJ202006002&uniplatform=OVERSEA&v=nKsHsMLriYD_W7DkDbihK5OaPOCu1h8STPgkLtkDOrtOdv00jN3QGHP0HhX3rJEo

