# Supplementary material for: How long can Chinese women work after retirement based on health level: Evidence from the CHARLS
Source: Front Public Health. 2023 Feb 27;11:987362. doi: 10.3389/fpubh.2023.987362 (PMC10009266; doi:10.3389/fpubh.2023.987362)
Supplement: Supplementary file 1 [file Table_1.docx]

**Appendix A**

**Table A1.** First principal component of PVW index by year.

| Health measure | 2011 | 2013 | 2015 | 2018 |
| --- | --- | --- | --- | --- |
| Physical limitation | 0.4105 | 0.4357 | 0.4149 | 0.4174 |
| Difficulty walking blocks | 0.3402 | 0.3439 | 0.3303 | 0.3253 |
| Difficulty climbing stairs | 0.3311 | 0.3447 | 0.3227 | 0.3256 |
| Difficulty lift/carry | 0.3198 | 0.3095 | 0.3143 | 0.2962 |
| Difficulty stoop/kneel/crouch | 0.3198 | 0.3257 | 0.3232 | 0.3292 |
| Difficulty getting up from chair | 0.2968 | 0.2681 | 0.2825 | 0.2593 |
| Difficulty with an ADL | 0.2769 | 0.2677 | 0.2582 | 0.2421 |
| Difficulty reach/extend arms up | 0.2588 | 0.2586 | 0.2628 | 0.2479 |
| SRH | 0.2276 | 0.2306 | 0.2284 | 0.2524 |
| Difficulty pick up a dime | 0.2242 | 0.1781 | 0.1989 | 0.1761 |
| Arthritis | 0.1275 | 0.1445 | 0.1695 | 0.1729 |
| Heart problems | 0.1150 | 0.1139 | 0.1420 | 0.1614 |
| High blood pressure | 0.1008 | 0.1104 | 0.1080 | 0.1393 |
| Hospital stays | 0.0986 | 0.0979 | 0.1141 | 0.1198 |
| Lung disease | 0.0793 | 0.0930 | 0.1069 | 0.1295 |
| Diabetes | 0.0775 | 0.0677 | 0.0959 | 0.1211 |
| Doctor visits | 0.0608 | 0.0734 | 0.0757 | 0.0854 |
| Psychological problems | 0.0421 | 0.0400 | 0.0601 | 0.0808 |
| Cancer | 0.0244 | 0.0098 | 0.0246 | 0.0284 |
| N | 8011 | 8034 | 8004 | 7888 |

**Table A2.** Definition and coding of variables.

| Variable | Definition |
| --- | --- |
| labor participation | Labor participation = 1, no labor participation = 0. |
| PVW index | Percentile scale from 1 to 100, with higher scores associated with better health. |
| SRH | Very Good =1, good = 2, fair = 3, poor = 4, very poor = 5. |
| CESD-score | Continuous variables, between 0 and 30.The higher the value, the lower the health level. |
| Physical Limit | No physical limits = 0, one physical limit = 1, more than one physical limit = 2**.** |
| ADL | Any ADL=1，no ADL=0. |
| IADL | Any IADL=1，no IADL=0. |
| Chronic disease | No chronic disease=0, Number of chronic disease is 1=1,Number of chronic disease is greater than 1=2 |
| Disability | With disability = 1, without disability = 0. |
| Psychiatric Condition | With psychiatric condition = 1, without psychiatric condition = 0. |
| Eyesight Poor | Yes=1, No=0. |
| Hearing Poor | Yes=1, No=0. |
| Fall down | Yes=1, No=0. |
| Fracture | Yes=1, No=0. |
| Smoke | Yes=1, No=0. |
| Drink | Yes=1, No=0. |
| Age | Categorical variable, age 45-74 years was assigned as a group every 5 years, for a total of 6 groups. |
| Marriage | Married =1, unmarried = 0. |
| Residence | Rural = 1, urban = 0. |
| Education | Illiteracy = 1, Elementary = 2, Middle and Above = 3. |
| Health insurance | With health insurance = 1, without health insurance = 0. |
